# Effects of A Brief Resonance Frequency Breathing Exercise on Heart Rate Variability and Inhibitory Control in the Context of Generalised Anxiety Disorder

**DOI:** 10.1007/s10484-025-09687-0

**Published:** 2025-02-09

**Authors:** David M. Spalding, Toni Ejoor, Xiaochang Zhao, Daniele Bomarsi, Martina Ciliberti, Cristina Ottaviani, Milan Valášek, Colette Hirsch, Hugo D. Critchley, Frances Meeten

**Affiliations:** 1 Department of Psychology, Institute of Psychiatry, Psychology and Neuroscience, King’s College London, London, UK; 2 Department of Psychology, University of Amsterdam, Amsterdam, The Netherlands; 3 Department of Psychology, Sapienza University of Rome, Rome, Italy; 4 Functional Neuroimaging Laboratory, IRCCS Santa Lucia Foundation, Rome, Italy; 5 Faculty of Architecture and Urbanism, Bauhaus University Weimar, Weimar, Germany; 6 Department of Neuroscience, Brighton and Sussex Medical School, University of Sussex, Brighton, UK; 7 Sussex Partnership NHS Foundation Trust, Worthing, UK; 8 Sussex Neuroscience, University of Sussex, Brighton, UK; 9 South London and Maudsley NHS Foundation Trust, Maudsley Hospital, Denmark Hill, London, UK; 10 School of Psychology, University of Sussex, Brighton, UK

**Keywords:** Generalised anxiety disorder, Worry, Heart rate variability, Resonance frequency breathing, Inhibitory control

## Abstract

Generalised anxiety disorder (GAD) is associated with cognitive and physiological symptoms including uncontrollable worry, inhibitory control deficits, and low heart rate variability (HRV). Literature linking HRV and inhibition in GAD is predominantly correlational. The present experiment investigated whether HRV has a causal role in maintaining inhibitory control. Participants (*N* = 135, 111 female) aged 18–37 reporting high levels of symptoms associated with GAD (GAD-7 scores ≥ 10; Penn State Worry Questionnaire scores ≥ 56) were assigned to an experimental or active control condition and completed baseline measures of HRV, respiration rate, and inhibitory control. The experimental condition completed resonance frequency breathing (RFB) training, and the control condition practiced breathing at their mean breathing rate before repeating the inhibitory control assessment. Participants also completed the breathing training before a behavioural worry task. The experimental condition was predicted to show increased HRV, alongside improved inhibitory control and better ability to stop worrying as compared to the control condition. HRV increased during the experimental condition, as compared to the control condition. However, there were no significant effects of RFB on inhibitory control or worry, or on HRV during the inhibitory control tasks. In conclusion, RFB can increase HRV in high GAD scorers, but further research is required to determine whether there is a relationship between increased HRV and symptoms of GAD in a single session experiment.

**Supplementary Information:**

The online version contains supplementary material available at 10.1007/s10484-025-09687-0.

Anxiety disorders are the most prevalent mental health disorder worldwide (Stein et al., 2017). Generalised anxiety disorder (GAD) is highly prevalent amongst the anxiety disorders (6.2% lifetime prevalence), second only to social anxiety disorder (Szuhany & Simon, 2022), and highly co-morbid with other anxiety conditions and major depressive disorder, with comorbidity prevalence ranging from 66.3 to 90.4% (Noyes, 2001; Wittchen, 1994). Key symptoms of GAD include excessive and uncontrollable worry, difficulty concentrating, and irritability. Somatic symptoms include muscle tension and feeling on edge, often accompanied by sleep disturbance (American Psychiatric Association, 2013).

Cognitive Behavioural Therapy (CBT) is the recommended first-line psychological treatment for GAD (National Institute for Health and Care Excellence; NICE, 2020). When considering treatment effects across the anxiety disorders, CBT is consistently associated with greater symptom reduction than waitlist, placebo, and no-treatment control conditions (Hedman et al., 2012; Hoffman & Smits, 2008; Otte, 2011). However, only 51–65% of those who receive CBT move into recovery (e.g., score below clinically indicative cut-offs on measures of anxiety after treatment; Springer et al., 2018). Pharmacological interventions, such as selective serotonin reuptake inhibitors and other antidepressant medications, are also commonly used in the treatment of GAD (NICE, 2020). However, research indicates that up to 38% of individuals experience cognitive and/or physiological side effects of antidepressants and the experience of side effects accounts for 23–36% of patients who discontinue treatment (Kelly et al., 2008). High levels of symptoms that do not meet the threshold for a diagnosis of GAD are twice as prevalent as diagnosed cases and can also have a debilitating effect on professional and social functioning, physical health, and overall quality of life (Haller et al., 2014; Kertz & Woodruff-Borden, 2011). It is thus crucial that we develop ways of augmenting the efficacy of current evidence-based treatments or develop alternative therapeutic approaches to improve treatment outcomes.

In order to augment existing treatments or develop novel treatment approaches, it is important to understand the cognitive and physiological mechanisms underpinning GAD. These mechanisms can then be examined as potential targets for treatment. Anxiety has been associated with systemic cognitive and physiological inflexibility. Cognitive inflexibility in anxiety is characterised by reduced executive control of attention (Eysenck et al., 2007). This is seen in impaired ability to inhibit distraction (e.g., difficulty concentrating on a task at hand), or withhold prepotent responses, or to flexibly shift attention between stimulus sets or tasks (Shi et al., 2019). This impaired attentional control is thought to underpin the uncontrollable worry experienced by people with GAD (Hirsch & Mathews, 2012). Uncontrollable worry is suggested to manifest as subvocalised, repetitive thoughts which demand capacity-limited attentional resources. This results in difficulty in effectively and efficiently attending to and processing information (Rapee, 1993; Wells, 1995). Physiological inflexibility is also evident at high levels of anxiety, in the form of self-reported autonomic nervous system dysregulation such as increased heart rate, hyperventilation, and increased perspiration, both at trait level and in response to stress (Barros et al., 2022; Hoehn-Saric & McLeod, 2000; McLeod et al., 1986). As compared with self-reports, objectively measured changes in physiology in individuals reporting high levels of anxiety have not been observed consistently (Hoehn-Saric & McLeod, 2000). For example, some research has found a diagnosis of GAD or sub-clinical high levels of trait worry to be associated with increased and/or less variable heart rate during resting-state measurements (e.g., Bair et al., 2021; Knepp et al., 2015; Thayer et al., 1996), whereas other studies have reported no significant resting state or ambulatory variance compared to participants reporting comparatively low levels of anxiety (e.g., Held et al., 2021; Hoehn-Saric, 1989; Hoehn-Saric et al., 2004; Levine et al., 2016). Associations between anxiety and physiology are more reliably observed during active worry. Here, people with a diagnosis of GAD and unselected samples all demonstrate inflexibility in physiological processes regulated by the autonomic nervous system, including increased and/or invariant heart rate (e.g., Dua & King, 1987; Hofmann et al., 2005; Pieper et al., 2007; York et al., 1987). Initial causal evidence therefore suggests that worry, over and above a diagnosis of an anxiety disorder, may exert a top-down influence on physiological responses. Indeed, worry is considered crucial in the maintenance of anxious psychopathology (Hirsch & Mathews, 2012).

The Neurovisceral Integration Model (Park & Thayer, 2014; Smith et al., 2017; Thayer & Lane, 2000, 2009) proposes that the neural networks underpinning executive function such as attentional control and emotion regulation are implicated in the control of cardiac autonomic function. This relationship is proposed to be bi-directional such that there is also an ‘up-stream’ effect of autonomic activity on prefrontal regulation (Mather & Thayer, 2018). This framework thus holds implications for understanding the mechanisms by which GAD and its symptoms may be maintained or reduced. Heart rate variability (HRV; the variation in the time intervals between successive heartbeats) is an accessible index of autonomic function. HRV is an index of vagal nerve modulation of the heart. The vagus nerve is responsible for physiological regulation (e.g., the control of inflammatory immune responses and heart rate responses amongst other visceral processes; Browning et al., 2017; Pavlov & Tracey, 2012). Higher vagal activity reflects greater physiological regulation via the parasympathetic nervous system, particularly the control of anxious sympathetic activation, such that higher HRV is—typically—an indicator of the ability to adapt appropriately to environmental and physiological stress (Lehrer, 2007). Under conditions of stress, sympathetic nervous system activity is increased, engendering the cognitive and physiological states associated with the ‘fight-or-flight’ response, while causing withdrawal of parasympathetic activity (Friedman, 2007). If vagal activity reflective of parasympathetic regulation is reduced, so too is control over autonomic hyperarousal and cardiovascular homeostasis (Porges, 2023). People with GAD or high levels of trait anxiety have reduced HRV (Makovac et al., 2016a; for reviews see Chalmers et al., 2014; Kemp & Quintana, 2013) and meta-analytic evidence shows that tonic HRV is lower in GAD compared to other anxiety disorders such as panic disorder (Wang et al., 2023).

The Neurovisceral Integration Model suggests that impaired physiological control as reflected in lower HRV may underpin cognitive inflexibility. The prefrontal cortex has a central role in both top-down inhibitory processes including the executive control of cognition (Cohen et al., 1997; Curtis & D’Esposito, 2003; Kane & Engle, 2002) and self-regulatory behaviour (Heatherton & Wagner, 2011), and also in receiving and transmitting visceral signals to and from the vagus nerve (Breit et al., 2018). The prefrontal cortex also exercises an inhibitory influence over sympathoexcitatory neural circuits including the amygdala, which is central to fearful and anxious responses, suggesting the possibility of a bottom-up influence of vagal control over emotionality (Thayer & Lane, 2009). The Neurovisceral Integration Model therefore presents an explanation for the maintenance of anxiety-related symptoms through bidirectional cognitive and physiological control processes. Specifically, physiological arousal and inflexibility may arise from anxious cognitions such as worry, but simultaneously physiological dysregulation may result in impaired ability to control or manage worry (e.g., Makovac et al., 2016a, 2016b). This perspective warrants further exploration specifically within the context of GAD, for which additional key diagnostic symptoms encompass combined and interactive cognitive, emotional, and somatic experiences.

Consistent with the Neurovisceral Integration Model, resting-state HRV is positively associated with executive function including measures of inhibitory control and sustained attention (Hansen et al., 2003, 2004; Ottaviani et al., 2019; see Forte et al., 2019 for a review; see Magnon et al., 2022 for meta-analytic evidence). Moreover, HRV is positively associated with inhibitory control over threatening stimuli (Hansen et al., 2009; Krypotos et al., 2011), which are typically less well inhibited at higher levels of anxiety (Bar-Haim et al., 2007; Cisler & Koster, 2010). Crucially, HRV appears to moderate associations between anxiety and cognitive function. At high levels of trait anxiety, ‘low’ HRV as determined by a median split of HRV values has been associated with less efficient inhibition of conflicting information for neutral stimuli (Ramírez et al., 2015). Relatively low-to-average HRV power was also associated with less efficient disengagement from threatening stimuli in participants reporting high levels of trait anxiety, with no difference in disengagement between those reporting relatively low or high levels of trait anxiety when HRV levels were above the average (Cocia et al., 2012). Evidence therefore suggests that both threat-related cognitive biases and deficits in ‘cold’ cognitive inhibition associated with anxiety may be influenced by underlying physiological inflexibility.

Considering the Neurovisceral Integrative perspective, a potential avenue to augmenting CBT outcomes is to explore whether physiological markers of anxiety, particularly reduced HRV, can be successfully modified to exert a positive bottom-up influence on broader GAD symptoms such as uncontrollable worry and cognitive inflexibility. A mechanistic approach that shows promise for increasing HRV is the practice of slow-paced breathing with and without biofeedback (Sevoz-Couche & Laborde, 2022). Slow-paced breathing in relation to HRV is often achieved by HRV biofeedback (HRVB) training. HRVB training requires the individual to breathe at a rate of approximately 5.5 or 6 breaths per minute (bpm), synchronising breath inhalation with heart rate increases and breath exhalation with heart rate decreases (e.g., Lehrer, 2013). A specific ‘resonance frequency’ breathing rate can be identified, which is the breathing rate ranging from approximately 4.5 to 7 bpm that results in the greatest increases in low-frequency HRV power. Breathing at resonance frequency maximises the baroreflex, responsible for controlling blood pressure, by increasing changes in blood pressure per unit of change in heart rate. When participants breathe at or around resonance frequency, HRV is also increased. There is evidence that engaging in resonance frequency breathing (RFB) over periods of two-to-50 weeks can reduce clinical symptoms and subclinical experiences of depression, anxiety and stress (see Blase et al., 2021 for a review; Goessl et al., 2017 for a meta-analysis).

While there is an established evidenced-based protocol for conducting HRVB training over a period of 5 weeks (Lehrer et al., 2013), increases to HRV are reliably observed during short periods (e.g., 5 min) of breathing training (e.g., Laborde et al., 2022a, 2022b; You et al., 2021). There is evidence that a single 5-min session of slow-paced breathing can improve state mood (Magnon et al., 2022; Mikosch et al., 2010). Evidence also suggests that benefits to HRV are co-occurring with state mood changes (Wells, 1995), and that breathing specifically at resonance frequency may also maximise the positive influence of slow-paced breathing on HRV (Steffen et al., 2017). By comparison, effects of slow-paced breathing/RFB on cognitive performance are less consistent, and warrant further exploration, particularly in the context of single-session breathing training protocols. Some studies have demonstrated improvement in attention and working memory measures from baseline to post-training (Bahameish & Stockman, 2024; Bonomini et al., 2020; Laborde et al., 2022a), or relatively improved cognitive performance following slow-paced breathing compared with other breathing manipulations (Liang et al., 2023). Other results, by comparison, have not demonstrated significant improvements from baseline following training (Hoffmann et al., 2019), or equivalent improvements in cognition across biofeedback and control groups (Sherlin et al., 2010). Other studies have demonstrated training-related improvements in executive function under stress (Prinsloo et al., 2011), or specifically in participants who report higher levels of in-the-moment stress (Blaser et al., 2023).

Single session HRVB training or slow-paced breathing has, however, seldom been employed with participants reporting clinical levels of anxiety symptoms, and who therefore might demonstrate lower levels of HRV and inhibitory control as compared with the general population. Furthermore, extant studies have been limited regarding sample size and the consistency with which stress and anxiety are measured. Prinsloo et al. (2011) found that participants who reported high life stress and completed 15 min of HRVB training made significantly fewer mistakes and faster responses in a modified Stroop task from pre-to-post-training, whereas a control group reporting high life stress did not see significant improvements in performance. Data from the same study indicated that the control group saw no change in HRV metrics during the Stroop task before and after HRVB training, whereas HRV was higher in the HRVB group following training (Prinsloo et al., 2013). A key limitation of the study design was that just nine participants were included in each group. Sutarto et al. (2013) meanwhile focused on participants presumed to have high levels of job-related stress and found significant improvements in Stroop performance within an HRVB group and not a control group. but the sample size was small (*N* = 17–19 participants per group). Sherlin et al. (2010) similarly focused on participants who self-reported high levels of stress and found that stress reactivity was enhanced following 15 min of HRVB training, but found that HRVB training did not benefit Stroop performance to a greater extent than watching physiological feedback without breathing instructions. RFB represents an unobtrusive and non-invasive means of targeting symptom reduction and improvements in cognitive and physiological flexibility in participants who report clinically indicative levels of anxiety symptoms, or who have already been diagnosed with a disorder. However, there is a need for further research to address a number of limitations in the existing literature regarding relationships between anxiety, physiology, and cognition.

## The Present Study

Anxiety is characterised by reduced cognitive and physiological adaptability, evidenced respectively by reduced inhibitory control over attention and reduced HRV. There is initial evidence that engaging in RFB can reduce anxiety, increase HRV and improve cognitive performance. Despite this, there is currently no research assessing the effectiveness of RFB as a tool for reducing symptoms and improving cognitive and physiological flexibility in participants reporting clinical levels of anxiety and worry. There has also been limited focus on defining the inhibitory cognitive processes which may be improved by breathing training and whether worry, as the key cognitive symptom of GAD, can be better inhibited or controlled as a result of training.

The present research sought to determine whether a single session of RFB training could improve executive inhibitory control—including the ability to control worry—in a sample reporting clinically indicative levels of GAD symptoms. Two groups of participants completed baseline measures of behavioural inhibitory control before undergoing breathing training. The experimental condition completed a resonance frequency assessment and five minutes of RFB. The control condition completed a sham breathing training equivalent where they were asked to breathe at approximately their mean breathing rate, determined at baseline. Each group then completed the inhibitory control tasks again, followed by top-up breathing training, before completing a final task in which they were asked to inhibit experimentally induced worry. Heart rate and respiration were measured at an initial baseline assessment, and then during completion of the inhibition tasks, breathing training, and worry inhibition task.

Our preregistered hypotheses were as follows:

1. As a manipulation check, we predicted higher low-frequency HRV (LF-HRV) and lower mean breathing rates in the RFB condition as compared with the control condition at each breathing training phase.
2. Regarding HRV as indexed through root mean square of successive differences (RMSSD) and high-frequency HRV (HF-HRV), we predicted there would be a significant two-way interaction between condition and time, whereby HRV is significantly higher in the RFB condition at time 2 (the post-breathing training task block) as compared with time 1 (the pre-breathing training task block) and as compared to the control condition.
3. Regarding cognitive outcomes, we predict a significant two-way time x condition interaction with participants in the RFB training condition demonstrating significantly better response inhibition ability across inhibition tasks: showing both a significant improvement from time 1 to time 2, and also showing significantly better performance than the control condition at time 2.
4. In the Stop Worry Task we predicted that participants in the RFB training condition would demonstrate better worry inhibition as compared to control condition participants.

## Method

The study commenced following approval from the King’s College London Health Faculties Research Ethics Subcommittee and the Institutional Review Board of the Department of Psychology, Sapienza University of Rome. All participants provided informed consent prior to their participation. The study was performed in accordance with the ethical standards as laid down in the 1964 Declaration of Helsinki and its later amendments.

### Experimental Design

The study was a two-arm (HRVB; control) randomized control experiment. Groups were stratified by gender. Randomization sequences was generated for female participants, male participants, and participants who responded with another gender category by a researcher (FM) who was not involved in data collection.

### Participants

Participants were recruited at two sites: King’s College London and Sapienza University of Rome. The sample comprised 135 participants. Participant demographic information at each site can be viewed in Table 1 and ethnicity information collected at each site can be viewed in Table 2. An a-priori power analysis using G*Power 3 (Faul et al., 2007) indicated the sample size was sufficient to detect a medium effect size (*d* = 0.5), using an alpha of 0.05 and power of 0.80. Further detail on the power calculation is in Supplement 1.

**Table 1:** Participant demographic information within overall sample and each condition

	Overall (N = 135)	Control (N = 69)	Experimental (N = 66)
*N* (% of sample)	135 (100%)	69 (51.1%)	66 (48.9%)
Age, *M* (*SD*)	24.2 (4.5)	23.7 (4.3)	24.7 (4.7)
Gender			
Female	111 (82.2%)	57 (82.6%)	54 (81.8%)
Male	18 (13.3%)	7 (10.1%)	11 (16.7%)
Non-binary/third gender	4 (3%)	3 (4.3%)	1 (1.5%)
Prefer to self-describe	2 (1.5%)	2 (2.9%)	0 (0%)
Prefer not to say	0 (0%)	0 (0%)	0 (0%)
BMI, *M* (*SD*)	23.3 (2.2)	22.0 (3.0)	22.3 (3.2)
Diagnosis of anxiety disorder	28 (20.7%)	14 (20.3%)	14 (22.2%)
SSRI use	2 (1.5%)	1 (1.5%)	1 (1.6%)

Percentages are calculated within groups. Due to rounding, percentages may not always total 100%

*BMI* body mass index, *SSRI* selective serotonin reuptake inhibitor, *M* mean, *SD* standard deviation

**Table 2:** Participant ethnicity information at each testing site

	Overall (*N* = 135)	Control (*N* = 69)	Experimental (*N* = 66)
London			
Asian/Asian British			
Bangladeshi	3 (2.2%)	1 (1.4%)	2 (3%)
Indian	8 (5.9%)	3 (4.3%)	5 (7.6%)
Pakistani	2 (1.5%)	0 (0%)	2 (3%)
Any other background	12 (8.9%)	6 (8.7%)	6 (9.1%)
Black/African/Caribbean			
African	3 (2.2%)	1 (1.4%)	2 (3%)
British	1 (0.7%)	0 (0%)	1 (1.5%)
Caribbean	2 (1.5%)	1 (1.4%)	1 (1.5%)
Any other background	0 (0%)	0 (0%)	0 (0%)
Mixed ethnicity			
Any other background	1 (0.7%)	0 (0%)	1 (1.5%)
White and Asian	1 (0.7%)	0 (0%)	1 (1.5%)
White and Black African	0 (0%)	0 (0%)	0 (0%)
White and Black Caribbean	0 (0%)	0 (0%)	0 (0%)
Other Ethnic Group			
Arab	3 (2.2%)	0 (0%)	3 (4.5%)
Any other ethnic group	1 (0.7%)	0 (0%)	1 (1.5%)
White			
English/Scottish/Welsh/Northern Irish/British	27 (20%)	11 (15.9%)	16 (24.2%)
Gypsy or Irish Traveler	0 (0%)	0 (0%)	0 (0%)
Irish	1 (0.7%)	1 (1.4%)	0 (0%)
Any other background	15 (11.1%)	10 (14.5%)	5 (7.6%)
Rome			
Black			
Italian	0 (0%)	0 (0%)	0 (0%)
Any other non-Italian origin	0 (0%)	0 (0%)	0 (0%)
White			
Italian	51 (37.8%)	31 (44.9%)	20 (30.3%)
Any other non-Italian origin	2 (1.5%)	2 (2.9%)	0 (0%)
Other Ethnicity			
Any other ethnic group	1 (0.7%)	1 (1.4%)	0 (0%)

Percentages are calculated within groups. Due to rounding, percentages may not always total 100%. Ethnicity categorisation in the UK was based on UK Government census guidelines for recording ethnicity

Inclusion criteria were (i) an age of between 18 and 40 years, and (ii) scoring ≥ 10 on the Generalised Anxiety Disorder Questionnaire (GAD-7; Spitzer et al., 2006) and ≥ 56 on the Penn State Worry Questionnaire (PSWQ; Meyer et al., 1990). Exclusion criteria, assessed by self-report, were (i) diagnosis of a depressive disorder, psychosis, emotionally unstable personality disorder/borderline personality disorder, or bipolar disorder, diagnosis of hypertension or heart disease, (ii) diagnosis of any other disease that might affect cardiovascular function, (iii) diagnosed or suspected cognitive impairments, (iv) substance use, (v) having a pacemaker installed, (vi) current use of drugs and medications that might affect cardiovascular function, (vii) psychotropic drugs, (viii) stimulants or recreational drugs, (ix) beta blockers, heart medication, bronchodilators, respiratory stimulants, pregabalin, antidepressants or anti-anxiety medications other than SSRIs, mood stabilizers, antipsychotic medication, and/or steroids, (x) currently pregnant or were pregnant within the previous 12 months, (xi) taking hormone replacement therapies, experiencing menopause or perimenopause symptoms, or (xii) moderate obesity (a BMI of ≥ 30). Participants were also excluded if they reported: having visual impairments that are not corrected by glasses or contact lenses, lacking proficiency in English, consuming more than 14 units of alcohol per week.

### Breathing Training and Physiological Recording

For all stages of RFB training and data recording, the software used was Biograph Infiniti Version 6.8.2.1 (Thought Technology Ltd., Quebec, Canada). Data was collected via a respiration belt and three-lead ECG sensors connected using the Biograph Procomp 5 Infiniti encoder.

#### Resonance Frequency Assessment

The unique resonance frequency for each participant in the RFB group was determined using the ‘broad resonance frequency’ assessment script of the Biograph Infiniti software. Participants were asked to breath in sync with a visually presented breathing pacer. Initially, participants were asked to breath at a rate of 7.0 bpm of two minutes. Breathing pace decreased by increments of 0.5 bpm every two minutes. The final breathing rate was 4.5 bpm. The breathing and heart rate signals were then visually inspected for artifacts within the Biograph software. Note, as research suggests that RFB rates range from 6.5 to 4.5 bpm (Shaffer & Meehan, 2020), we modified the default programme to remove the initial 7.5 bpm pacer and treated the 7.0 bpm pacer as a period for participants to acclimatise to slow-paced breathing. Artifacts were removed and the RFB rate for that individual was then determined as the breathing rate—automatically calculated within Biograph—which corresponded with the highest LF-HRV power. Occasionally, the respiration belt was sensitive to detecting false respiration cycles which in some cases artificially increased participants’ automatically calculated breathing rate. In these cases, we designated the pacer rate which corresponded to the greatest increase in low frequency HRV as the participants’ resonance frequency.

#### Experimental Condition (RFB Training)

Participants were asked to breathe at their resonance frequency breathing rate for a period of 5 min in a first training period, and again for 5 min in a second training period.

#### Control Condition (Mean Breathing Rate Training)

Here, the aim was to provide an active control condition that maps closely onto the resonance frequency assessment and training without manipulating HRV. To mimic the resonance frequency assessment, the mean breathing rate from the baseline assessment was taken as each participant’s natural breathing rate. From this rate, participants were asked to breath at three increments of 0.2 bpm above their mean breathing rate, and two increments of 0.2 below their mean breathing rate. Participants followed the pacer at these rates for two minutes at each increment. Therefore, if a participant’s natural mean breathing rate was 12 bpm, then they would begin the resonance frequency equivalent condition by breathing at 12.6 bpm, decreasing in increments of 0.2 bpm every two minutes until they were breathing at 11.6 bpm. Each individual’s mean natural breathing rate was used as the target breathing rate during the biofeedback-equivalent stage, with participants asked to focus on breathing in time with a pacer presented on screen for equivalent durations to the RFB training condition.

#### HRV Data Processing

HRV analyses were conducted in Kubios version 4.1 (Tarvainen et al., 2014). Raw ECG data was entered for calculation of R-R intervals and variability indices. Kubios detects R-waves automatically, but all data were visually inspected for missing or incorrect beats which were then manually added or removed, respectively. We interpreted and identified true R-peaks as those which were approximately the shape of the combined P-wave, QRS complex, and T-wave which are typical of a single heartbeat. False R-peaks were interpreted and identified as those that were not clearly identifiable as the approximate R-peak shape. Then, abnormal beat intervals were corrected using Kubios’ validated automatic algorithm which detects abnormal values from an observed time series of different RR values within the signal (Lipponen & Tarvainen, 2019). Via the automatic correction algorithm, ectopic and overly long or short beats are corrected by replacement with interpolated values. Missing beats are corrected with the insertion of a new R-wave occurrence in the signal and extra beats are conversely corrected by removal of the R-wave occurrence, before the time series is recalculated. RMSSD, HF-HRV, LF-HRV indices were extracted from Kubios, including the log-transformed HF-HRV and LF-HRV values. The log-transformed RMSSD value was subsequently calculated in R and SPSS prior to data analyses. RMSSD and HF-HRV are highly correlated and index parasympathetic nervous system activity and vagally mediated HRV. Both indexes were investigated, however, HF-HRV is influenced by respiration rate while RMSSD is less affected by respiration (Shaffer & Ginsberg, 2017). It was therefore important to determine whether patterns of findings were consistent across these indices. The LF-HRV index was used to determine the efficacy of the breathing manipulation. LF-HRV reflects vagally mediated changes in HRV specifically during slow-paced breathing (Shaffer & Ginsberg, 2017) and was therefore expected to be significantly increased under resonance frequency breathing as compared with mean-rate breathing.

### Behavioural Tasks

#### Stimulus Inhibition

A computerised version of the colour-word Stroop task (Stroop, 1935) was used as a measure of attentional inhibition (Cothran & Larsen, 2008). The version of the task available on the Inquisit library was run on Inquisit Lab 6 (Millisecond Software, 2020). In the task, participants are presented with one of three stimuli in the centre of the screen and are asked to indicate, via keypress, the colour of the stimuli being presented; colour words presented in the same colour font as the word (congruent condition), colour words presented in a different colour font from the word (incongruent condition), and coloured rectangles (control condition). The colours used were red, green, blue, and yellow. Participants were asked to name the colour being shown as fast and accurately as possible, as they were being timed. In the incongruent condition, participants must inhibit the meaning of the word in order to correctly identify the colour the word was presented in. Responding with the word itself, rather than the colour it is presented in, indicates a failure to inhibit the word. There were 84 experimental trials. Each colour was tested equally within each condition, across seven repetitions (four colours x three conditions x seven repetitions). Stimuli stayed on the screen until the participant responded. Inter-trial intervals lasted 200 ms, and error feedback in the form of a red cross presented at the bottom of the screen was presented for 400 ms. The overall time to complete the task was approximately 5 min. Colour-word Stroop performance was indexed as an inverse efficiency score: (mean RT of correct responses/[1—error rate]) and an interference index based on the inverse efficiency score (incongruent inverse efficiency scores—congruent inverse efficiency scores) (Riedel et al., 2021; Wolff et al., 2016). Higher positive scores indicate worse inhibition of incongruent stimuli. For the Rome subsample, colour words were translated to Italian by the Sapienza University research team.

#### Emotional Inhibition

A computerised version of the emotional Stroop task available on the Inquisit library and run using Inquisit Lab 6 was modified to assess the inhibition of stimuli of varying emotional valence. Participants were asked to indicate via keypress the colour of the word being presented. In this case, words were drawn from a list of emotionally negative words, or emotionally neutral words. Presentation times were the same as in the colour-word Stroop task. We followed the recommendations of Ben-Haim et al. (2016) during design and programming of the emotional Stroop in order to maximise the likelihood of observing emotional Stroop effects. To avoid repetition of words, each word was only presented once and therefore the colour in which each word was presented was randomised. Each colour was presented four times per block. Neutral and negative stimuli were presented in discrete blocks. The neutral block was always presented before the negative block, and there was no gap between blocks. Different sets of negative and neutral words were used for each administration of the emotional Stroop. Words were taken from Hsieh and Sharma (2019). In their study, there were two sets of 20 negative words and six sets of 20 neutral words matched for frequency of use and length. The negative word sets were matched for valence and arousal, as were the neutral words. We used each set of negative words (one per administration of the emotional Stroop) and the first two lists of neutral words (one per administration of the emotional Stroop). The overall time to complete the task was approximately 2 min. Like the colour-word Stroop, emotional Stroop performance was indexed via an inverse efficiency score and an interference score (negative word inverse efficiency scores—neutral word inverse efficiency scores). Higher positive scores indicate worse inhibition of negative stimuli. For the Rome subsample, negative and neutral words were translated to Italian by the Sapienza University research team.

#### Response Inhibition

The Sustained Attention to Response Task (SART; Robertson et al., 1997) was used to measure response inhibition. The version used was available on the Inquisit library and run using Inquisit 6. In the SART, participants are asked to view a sequence of single digits (ranging from 1–9) of varying font sizes, presented in the centre of the screen. Each digit is presented for 250 ms before being replaced by a mask (a circle with a cross in the centre), which is presented for 900 ms. The period from digit-to-digit onset is therefore 1150 ms. Participants are asked to press the spacebar each time a digit other than ‘3’ appears on the screen. When ‘3’ appears, the participant is asked to make no response. Each digit is presented 25 times, such that 88.88% of trials are ‘go’ trials, and the remaining 11.11% of trials are ‘no-go’ trials, with the increased frequency of ‘go’ responses often resulting in poorer inhibition of prepotent ‘no-go’ error responses. The overall time to complete the task was approximately 4 min. SART performance was measured via a skill index (No-Go accuracy/mean Go response time), where No-Go accuracy = ([total No-Go trials—commission errors]/total No-Go trials)*100 (Hallion et al., 2020), with higher scores indicating better performance.

#### Inhibition of Worry

The Stop Worry Task (SWT; Hirsch lab^1^) was used to assess participants’ ability to stop worried thoughts. In the task, participants identify a topic that they were particularly worried about, and are asked questions on the topic intended to facilitate worry. Participants are then left to worry for a 2-min period. A tone then sounds indicating that participants should stop worrying. A prompt (‘Thoughts?’) appears on the screen, and participants are asked to indicate via keypress whether they have stopped worrying or not. This ‘stop worry’ phase of the task lasted 5 min, with participants prompted to indicate whether they have stopped worrying via presentation of the tone and prompt every 10 s. Pilot work on this task has found that people with higher trait worry (as measured by a self-report questionnaire) show more difficulty in disengaging from worry (Mackintosh, 2017), suggesting this behavioural task reflects self-reported worry style. A Stop Worry Index is calculated as the total number of non-negative thoughts/total number of responses, with higher scores indicating better ability to stop worrying.

### Self-Report Measures

#### GAD

The GAD-7 (Spitzer et al., 2006) is a 7-item measure designed as a screening tool for GAD symptoms over the two weeks prior to completing the scale. It is a reliable and valid measure of anxiety in the general population, with Cronbach's alpha values between 0.89 and 0.92 (Löwe et al., 2008; Spitzer et al., 2006). Items are scored on a 4-point Likert scale from 0 (“Not at all”) to 3 (“Nearly every day”). Scores range from 0–21, scores ≥ 10 indicate ‘moderate’ anxiety and ≥ 15 ‘severe’ anxiety with a threshold of 10 having 89% sensitivity and 82% specificity for a GAD (Kroenke et al., 2007).

#### Worry

The Penn State Worry Questionnaire (PSWQ; Meyer et al., 1990) is a 16-item measure of trait worry experienced in the two weeks prior to completing the questionnaire which has been shown to be reliable in both clinical and nonclinical samples (Brown et al., 1992; Korte et al., 2016), Cronbach’s *α* = 0.93 (Brown et al., 1992). Items are scored on a 5-point Likert scale from 1 (“Not at all typical of me”) to 3 (“Very typical of me”). Scores range from 16–80. Scores ≥ 56 have been used to identify ‘high worriers’, being one standard deviation below the mean of a sample diagnosed with GAD (Molina & Borkovec, 1994).

#### Depression

The Personal Health Questionnaire Depression Scale (PHQ-8; Kroenke et al., 2009) is an 8-item measure which assesses the severity of depression symptoms. Items are scored on a 4-point Likert scale ranging from 0 (“Not at all”) to 3 (“Nearly every day”). The PHQ has demonstrated high internal consistency, Cronbach’s *α* = 0.87 (de la Torre et al., 2023).

#### State Mood, Worry, and Thought Patterns

Visual analogue scales (VAS) were used between biofeedback and cognitive task blocks to indicate present levels of worry, mood and repetitive intrusive thoughts during the experimental session. VAS have been extensively validated for assessing mood and psychological states, showing strong reliability and correlations with more complex measures (Nyenhuis et al., 1997; Stern et al., 1997). Participants were asked to indicate how much either “Right now” or “in the tasks [they] just completed” how much each of the following statements applied to them, rated on a sliding scale from “Not at all” to “Very much”. Descriptive statistics for all the VAS mood measures are reported in Table S1. VAS questions were as follows:

- How much are/were you distracted by your thoughts (i.e. past memories, future worries, personal problems)?
- How much are/were these thoughts going through your mind again and again?
- How much are/were these thoughts coming to your mind without you wanting them to?
- How much do/did you feel anxious?
- How much do/did you feel worried?
- Right now, how much do/did you feel happy?
- Right now, how much do/did you feel sad?

Additional baseline measures of the Perseverative Thinking Questionnaire (PTQ; Ehring et al., 2011) and the Trait subscale of the State-Trait Inventory for Cognitive and Somatic Anxiety (STICSA-T; Ree et al., 2008) were collected but are not reported on in the current study.

### Procedure

**Fig. 1:**
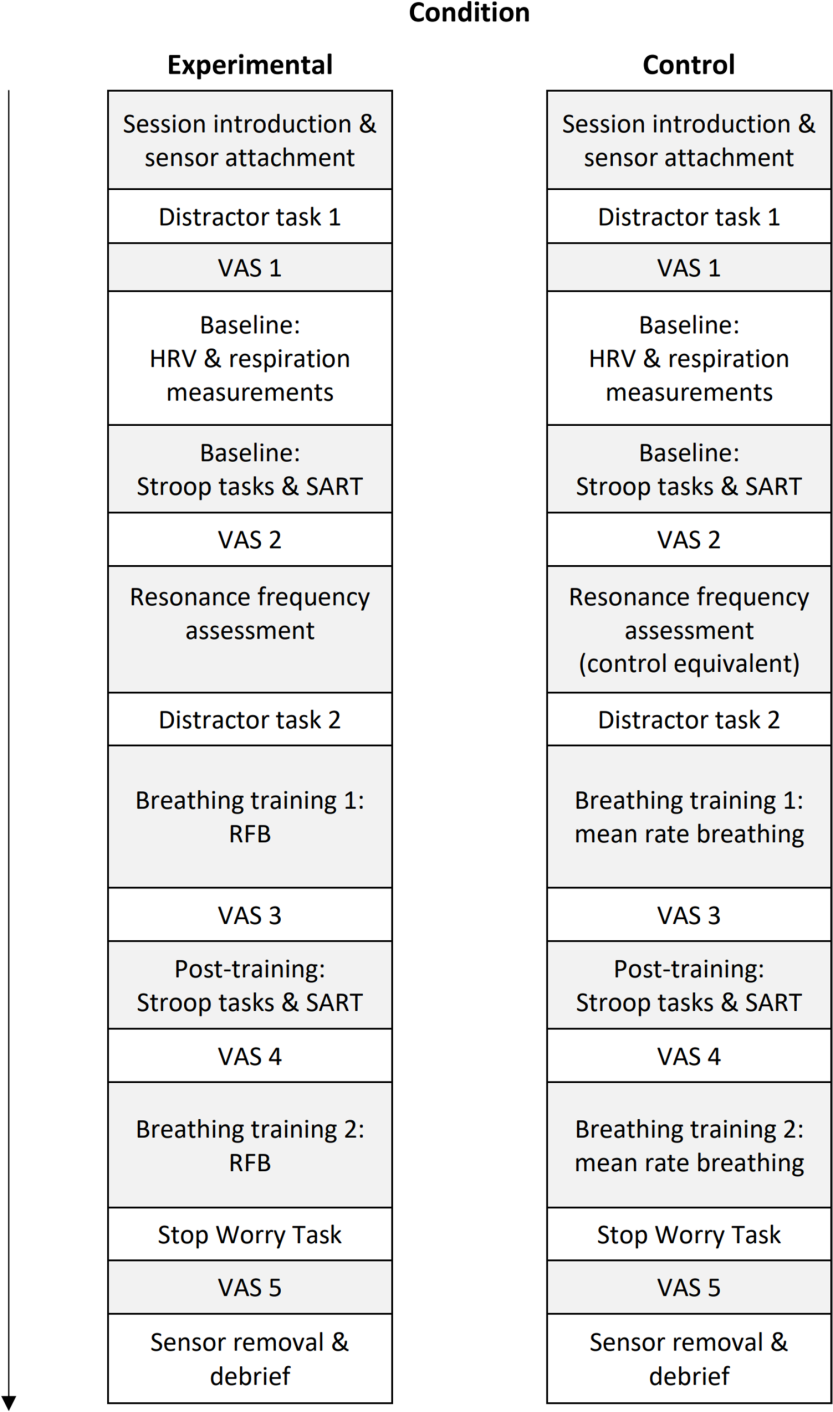
Experimental procedure. *HRV* heart rate variability, *RFB* resonance frequency breathing, *SART* sustained attention to response task, *VAS* visual analogue scales

An overview of the experimental protocol is provided in Fig. 1. Participants completed consent and demographic information prior to the GAD-7, PSWQ, STICSA, PHQ-8, and PTQ (in that order) via an online questionnaire within 24 h prior to arriving at the lab. Upon arriving at the lab, participants were equipped with the ECG and respiration sensors. Participants then completed a short distractor task to facilitate acclimatisation to the testing environment. A baseline measure of HRV was then taken. HRV was then measured during the subsequent breathing training stages and executive function task blocks. Visual analogue scales were completed between each block.

Following initial baseline measures of self-report questionnaires and resting state HRV, participants completed the Stroop tasks and SART. The RFB training/comparative control training then took place. In the RFB training condition, participants were first familiarised with the breathing pacer and undertook the resonance frequency assessment. This frequency informed the target breathing rate in the subsequent RFB training phases. In the control condition, participants were also familiarised with the breathing pacer and completed the comparative control assessment equivalent. All participants completed a distractor task to provide a break between the resonance frequency assessment stage and the subsequent breathing training period. Participants then completed the Stroop tasks and SART a second time (time 2). A final top-up session of breathing training was then administered, after which participants completed the SWT. Participants were then thanked and debriefed.

## Results

All analyses to test the main study hypotheses were conducted in R version 4.4.2 (R Core Team, 2021). Descriptive statistics were calculated in R and SPSS version 29 (IBM Corp., 2023). Note that the sample size varies in each analysis depending on missing data.

### Data Checks

For the colour-word Stroop task, all participants met the cutoff criterion of 50% accuracy and so none were removed from the analysis. A total of 216 trials (113 at time 1; 103 at time 2) from 112 participants were excluded from the final task score calculation due to reaction time being longer than mean + 3 SD, calculated per participant, timepoint, and Stroop condition. The maximum number of trials excluded for a given participant were two trials at time 1 and three trials at time 2.

For the emotional Stroop task, all participants met the cutoff criterion of 50% accuracy and so none were removed from the analysis. A total of 136 trials (72 at time 1; 64 at time 2) from 88 participants were excluded from the final task score calculation due to reaction time surpassing the cutoff criterion. The maximum number of trials excluded for a given participant were two trials at time 1 and two trials at time 2.

Examination of Q-Q plots and histograms indicated that RMSSD, LF-HRV and HF-HRV values were skewed, and therefore we conducted analyses on log-transformed values. We used the standard *p* < 0.05 inference criteria to determine statistical significance for all analyses.

### Control Variables

A series of preliminary analyses were conducted to determine whether any control variables should be included in the main analyses. We assessed whether the experimental and control group differed in diagnoses of an anxiety disorder, use of hormonal contraceptives, over-the-counter medication, SSRIs, alcohol, caffeine, nicotine, recreational drugs, previous night’s sleep quality or self-reported fitness levels varied by anxiety group. Full results can be viewed in Supplement 3 and accompanying descriptive statistics in Table S3. There were no significant differences observed, all *p* > 0.16, all *t* < 1.08.

### Baseline Self-Report Measures

Participants completed validated self-report measures (see Table 3). Participants reported in the higher range of moderate anxiety as measured by the GAD-7 (where 10–14 is classed as moderate and 15–21 is severe anxiety; Spitzer et al., 2006). Participants reported in the severe range for depression on the PHQ-8 (Kroenke et al., 2009). Correlations (see Table 4) indicated significant positive relationships between PSWQ and GAD-7 scores and between GAD-7 and PHQ-8 scores, but no other significant relationships between baseline assessments.

**Table 3:** Baseline self-report questionnaire and RMSSD data by condition

		Control condition				Experimental condition				
	*N*	Mean	*SD*	Min	Max	*N*	Mean	*SD*	Min	Max
PSWQ	66	67.3	5.2	57	80	63	68.5	5.2	56	80
GAD-7	66	14.2	2.8	10	20	63	14.1	2.9	10	21
PHQ-8	68	14.6	6.4	2	29	66	13.4	5.8	0	26
RMSSD	68	37.0	28.3	3.3	167.9	61	40.6	21.7	11.0	123.9
RMSSD (log)	68	1.5	0.3	0.5	2.2	61	1.6	0.2	1.0	2.1

**Table 4:** Spearman Correlations Between RMSSD at baseline (log), PSWQ, GAD-7, and PHQ-8 scores

	1	2	3	4
1. PSWQ	–			
2. GAD-7	.38*	–		
3. PHQ-8	.06	.28**	–	
4. RMSSD (log)	.03	.08	− .1	–

^*^p < .001, **p = .001

### Manipulation Checks

To assess the efficacy of the breathing manipulation (i.e., whether RFB successfully increased HRV compared with breathing at approximately individuals’ mean breathing rate), independent samples *t*-tests were conducted to determine whether the groups significantly varied in LF-HRV and mean breathing rate during each paced breathing exercise (see Table 5 for mean breathing rate by condition). As expected, the experimental group had a significantly slower breathing rate than the control group during the first breathing exercise, *t*(132) = 24.57, *p* < 0.001, *d* = 4.24 and also during the second breathing exercise, *t*(129.63) = 25.05, *p* < 0.001, *d* = 4.23. The experimental group also had significantly higher LF-HRV than the control group during the first breathing exercise, *t*(131) = −14.44, *p* < 0.001, *d* = 2.52, and during the second breathing exercise *t*(128) = −13.01, *p* < 0.001, *d* = 2.31 We can also see that in the RFB group, participants’ mean breathing rate was at approximately 6 bpm with the SD being at 1.8 indicating that for the majority of time participants were breathing within the resonance frequency range. This suggests that our breathing pace and HRV manipulations were successful.

**Table 5:** Breathing rate (breaths per minute) and log transformed low-frequency heart rate variability (LF-HRV (log) at baseline and in each paced breathing exercise

	Control condition					Experimental condition				
	*N*	Mean	*SD*	Min	Max	*N*	Mean	*SD*	Min	Max
Breathing rates										
Baseline	69	15.4	3.7	8.2	25.6	65	14.0	4.1	5.7	26.8
Breathing exercise 1	69	14.6	2.1	8.6	18.4	65	6.1	1.9	4.0	13.2
Breathing exercise 2	69	14.5	2.2	8.8	19.0	65	6.0	1.8	4.0	12.2
LF-HRV (log)										
Baseline	68	6.4	1.1	3.6	8.8	61	6.7	1.0	4.4	9.5
Breathing exercise 1	68	6.5	0.9	3.3	8.3	65	8.9	1.0	5.9	11.0
Breathing exercise 2	67	6.7	0.9	3.8	8.5	63	8.9	1.0	6.0	11.1

### Main Analyses

To assess change in inhibitory control and HRV over time and between groups, linear mixed models were constructed. For each model, the fixed-effects structure included the effect of condition (experimental, control), the effect of time (pre-breathing training 1, post-breathing training 1), and their interaction. A random intercept was specified per participant, nested within condition. However, the random intercept for the emotional Stroop model was 0, and therefore an ordinary least squares (OLS) regression was used to assess whether the change score from first to second administration of the emotional Stroop varied by group. To assess effects of the breathing training on Stop Worry Task performance, a linear regression was conducted, to assess the association between condition (experimental, control) and the Stop Worry Index.^2^

#### HRV During Inhibitory Control Tasks

Note that because the pattern of significant main effects and interactions was similar across for both HRV indices in both the colour-word Stroop task and SART, we report only the Stroop results here for brevity. We did not analyse the HRV data for the emotional Stroop, given the short duration of the task. RMSSD and HF-HRV values for each colour-word Stroop task within each group can be viewed in Table 6.

**Table 6:** RMSSD (log) and HF-HRV (log) during each colour-word Stroop task

		Control					Experimental				
	Time	*N*	Mean	*SD*	Min	Max	*N*	Mean	*SD*	Min	Max
C-W Stroop RMSSD (log)	1	68	1.5	0.2	0.7	2.0	62	1.6	0.2	1.1	2.2
	2	68	1.6	0.2	1.0	2.1	62	1.6	0.2	1.2	2.2
C-W Stroop HF-HRV (log)	1	68	5.9	1.2	2.3	8.6	62	6.2	1.2	3.6	9.0
	2	66	6.2	1.2	2.8	8.7	62	6.3	1.2	4.0	9.5

Parameter estimates for each linear mixed model including RMSSD and HF-HRV as outcomes in the colour-word Stroop task can be viewed in Table 7. There were main effects of time, whereby RMSSD was higher during the second colour-word Stroop task (*M* = 1.6, SD = 0.2) than the first task (*M* = 1.5, *SD* = 0.2), and HF-HRV was also higher during the second task (*M* = 6.3, *SD* = 1.2) than the first task (*M* = 6.0, *SD* = 1.2). There was also a significant interaction between condition and time, whereby HF-HRV significantly increased from the first colour-word Stroop to the second colour-word Stroop in the control condition to a greater extent than in the experimental condition. Descriptive statistics for HRV values during the SART can be viewed in Table S3. Results for analyses including SART HRV values are presented in Table 4.

**Table 7:** Parameter estimates for each linear model including RMSSD (log) and HF-HRV (log) during each colour-word stroop administration

	95% Confidence intervals						
	*B*	*SE*	*df*	*t*	*p*	*LLCI*	*ULCI*
RMSSD (log)							
Intercept	3.50	0.06	149.94	54.46	< .001	3.38	3.63
Condition	0.10	0.09	151.09	1.06	.290	− 0.08	0.28
Time	0.20	0.04	124.76	5.67	< .001	0.13	0.27
Condition × time	− 0.08	0.05	125.10	− 1.56	.120	− 0.18	0.02
HF-HRV (log)							
Intercept	5.86	0.15	154.00	40.32	< .001	5.57	6.14
Condition	0.32	0.21	155.32	1.53	.128	− 0.09	0.73
Time	0.41	0.09	124.94	4.72	< .001	0.24	0.58
Condition x Time	− 0.28	0.13	125.33	− 2.20	.030	− 0.53	− 0.03

‘Intercept’ represents estimated values for control group in first Stroop task. ‘Condition’ represents estimated difference between experimental and control condition in Stroop task, ‘Time’ represents estimated change in control group from first Stroop task to second Stroop task, ‘Condition × Time’ represents the difference in the change from the first to second Stroop task between the experimental and control condition

#### Inhibitory Control Performance Outcomes

Descriptive data for inhibitory control task performance can be viewed in Table 8.

**Table 8:** Performance on inhibitory control tasks by condition and timepoint

		Control					Experimental				
	Time	*N*	Mean	*SD*	Min	Max	*N*	Mean	*SD*	Min	Max
Colour-word stroop	1	69	291.3	214.4	8.2	1042.0	65	381.8	339.1	− 59.2	2407.0
	2	69	232.0	195.1	− 163.9	782.8	65	317.4	270.2	− 0.1	1261.7
Emotional stroop	1	69	25.9	267.9	− 1423.8	809.6	65	171.8	326.2	− 456.6	1549.4
	2	69	57.7	160.8	− 638.0	338.3	65	72.8	120.0	− 194.1	403.9
SART	1	68	13.6	4.5	0.0	23.5	60	13.8	4.4	2.8	22.0
	2	68	14.4	4.6	0.0	23.3	60	14.8	4.1	4.8	22.6

Parameter estimates for each linear mixed model including SART and colour-word Stroop indices as outcomes can be viewed in Table 9. There were no significant effects of condition, time, or their interaction on the SART skill index. For the colour-word Stroop, there was a main effect of time, but the main effect of condition and the interaction were not significant. The interference score was significantly lower at the post-breathing exercise administration of the task (*M* = 273.4, *SD* = 237.5), as compared with the pre-breathing exercise administration (*M* = 335.2, *SD* = 284.5). This indicates that both groups were significantly better at inhibiting interference at the second administration of the task compared to the first.

**Table 9:** Parameter estimates for each linear model including the SART skill index and colour-word stroop inverse efficiency scores as outcomes

	95% Confidence Intervals						
	*B*	*SE*	*df*	*t*	*p*	LLCI	ULCI
SART skill index							
Intercept	13.60	0.54	180.04	25.39	< .001	12.56	14.65
Condition	0.16	0.78	180.04	0.21	.837	− 1.37	1.69
Time	0.82	0.46	126.00	1.79	.076	− 0.08	1.72
Condition × time	0.22	0.67	126.00	0.32	.758	− 1.10	1.53
CW stroop inverse efficiency							
Intercept	291.35	26.48	213.08	11.00	< .001	239.61	343.08
Condition	58.86	38.17	213.08	1.52	.125	− 15.72	133.44
Time	− 59.36	27.03	131.00	− 2.20	.030	− 112.31	− 6.41
Condition x Time	11.76	38.96	131.00	0.30	.763	− 64.57	88.10

‘Intercept’ represents estimated values for control condition in first task administration, ‘Condition’ represents estimated difference between experimental and control condition in first task administration, ‘Time’ represents estimated change in control condition from first task administration to second task administration, Condition x Time represents the difference in the change from first to second task administration between the experimental and control conditions

For the emotional Stroop, there was no significant effect of condition on change in emotional interference from the first administration of the task to the second, *F*(1, 128) = 1.12, *B* = 47.86, *SE* = 45.18, *t* = 1.06, *p* = 0.291.

#### Stop Worry Task

Descriptive data regarding the proportion of time participants reported having negative thoughts when asked to stop worrying are presented in Table 10.

**Table 10:** Proportion of non-negative thoughts reported during the stop worry task

Group	*N*	Mean	*SD*	Min	Max
Control	69	0.5	0.3	0	1.0
Experimental	65	0.5	0.2	0	0.9

There was no significant association between condition allocation and ability to disengage from worry, *F*(1, 132) = 0.28, *B* = 0.03, *SE* = 0.05, *t* = 0.53, *p* = 0.595. Post task assessment of self-reported engagement in worry during the worry phase showed that both conditions reported worrying for the majority of the worry phase. The control condition reported worrying 80.0% of the time (*SD* = 22.0) and the experimental condition reported worry 81.3% of the time (*SD* = 16.5).

## Discussion

The present experiment assessed whether a brief RFB exercise can significantly increase HRV and improve inhibitory control in participants reporting clinically indicative GAD symptoms. We sought to advance the field by adopting a conservative approach to statistical power as compared with other between-groups, single-session research. Furthermore, we asked participants to breathe at their own individual resonance frequency as opposed to a generic slow-paced breathing rate, which may maximally increase HRV (cf. Steffen et al., 2017). We also sought to employ an active control breathing condition which was generally comparable with the RFB procedures aside from the breathing pace.

We predicted that RFB would increase heart rate variability, specifically LF-HRV, thus confirming the efficacy of our experimental manipulation and serving as the basis for our key hypotheses. Our first key hypothesis was that those in the experimental condition, who completed RFB training, would show improved inhibitory control from pre- to post- training, as compared with the control condition, who breathed at their approximately their mean breathing rate. Our second key hypothesis was that the experimental condition would show better self-reported ability to stop worrying on the Stop Worry Task, following the breathing training, as compared with the control condition. While RFB did increase LF-HRV compared to the control condition as expected, our key hypotheses were not supported. Both the experimental and control groups showed significantly more efficient inhibition of incongruent stimuli in the second colour-word Stroop task than in the first. However, the experimental group did not improve to a significantly greater extent than the control group, and therefore group allocation did not explain the improvement in scores. No significant differences in emotional Stroop or SART performance were observed in the RFB group from pre-to post- training, nor did their performance significantly differ from the control group following the training. Furthermore, there were no significant differences in HRV indices (RMSSD and high-frequency heart rate variability, or HF-HRV) during the first and second administrations of the inhibitory control tasks. There was also no effect of RFB on a behavioural worry task indicating that increasing HRV did not result in improved ability to disengage from state worry.

The present inhibitory control outcomes contribute to a generally mixed literature as to the effect of single session RFB or slow-placed breathing on cognition. Evidence for improvements in executive function and related cognitive domains following short administrations (2–20 min) of slow-paced breathing or biofeedback training is varied. Where some studies show improved executive function associated with slow-paced breathing (Bahameish & Stockman, 2024; Bonomini et al., 2020; Laborde et al., 2022a; Liang et al., 2023), others suggest no specific benefit associated with breathing manipulations (Hoffman et al., 2019; Sherlin et al., 2010). Importantly, where improvements in cognitive performance have been observed in slow-paced breathing versus control groups, the relationship has not been mediated by a corresponding increase in HRV indices (Baheimish & Stockman, 2024; Laborde et al., 2022a), suggesting that improved performance did not arise specifically as a result of state changes to HRV.

Our findings make a unique contribution to this existing literature in suggesting that individuals reporting clinically indicative levels of GAD symptoms do not demonstrate cognitive benefits immediately following RFB. One explanation for this outcome is that high GAD scorers, at baseline, should typically be experiencing limited cognitive and physiological flexibility. Excessive, uncontrollable worry is the cognitive hallmark of GAD, attracting and depleting available cognitive resources and contributing to the maintenance of symptoms (Hirsch & Mathews, 2012; Ruscio & Borkovec, 2004). GAD and high levels of self-reported anxiety are also negatively associated with HRV (Chalmers et al., 2014; Cheng et al., 2022; Kemp & Quintana, 2013). It may therefore be assumed that cognitive and physiological adaptability were already reduced within our sample. The relatively short administrations of RFB in the present study may not have been sufficient to mitigate these existing deficits in inhibitory control and HRV indices.

The efficacy of our experimental manipulation provides context for the non-significant changes in inhibitory control presently observed. Considering the physiological effects of the breathing manipulation between groups, RFB did result in an increase in LF-HRV during breathing practice, whilst breathing at approximately mean rates did not. This confirms our chosen manipulation of HRV was effective in inducing in-the-moment changes to HRV. Increases in RMSSD and both LF and HF frequencies during 2–5 min of slow-paced and/or RFB periods have been observed consistently in the literature (Bonomini et al., 2020; Laborde et al., 2022a; MacKinnon et al., 2013; Steffen et al., 2017; Strausse-Blasche et al., 2001; You et al., 2022, 2024). However, a lack of significant increases in RMSSD or HF-HRV between executive function task administrations in the present study suggests that change in HRV was not sustained when participants subsequently engaged in a cognitively demanding task. This is consistent with previously literature which shows that varying short periods of breathing training (5–20 min) result in similar increases to HRV during training, but that increases are not maintained during subsequent spontaneous breathing (You et al., 2021) or task performance (Laborde et al., 2022a).

The acute effect of the breathing training on HRV may explain the lack of significant effects of the breathing manipulation on inhibitory control. From the Neurovisceral Integrative perspective, increased parasympathetic control and vagal activity, as indexed by HRV, should correspond to increased cognitive control (Thayer & Lane, 2009). In the present study, if the index of parasympathetic control and vagal activity (in this case, HRV) was not significantly different across task pre- and post-training administrations, the mechanism by which cognitive control was expected to improve was not ‘activated’ to any greater extent during performance of the second inhibitory control tasks. That is, HRV was not significantly increased at the second task administration compared to the first, and therefore effects on cognition may not be expected. An important next step in determining the mechanistic influence of vagal activity as indexed by HRV on cognitive outcomes—specifically in the context of high GAD symptoms—would be to determine how inhibitory control abilities might vary during active stimulation of the vagus nerve. Given that breathing training requires attentional engagement that could distract from focus on cognitive tasks, future research could employ passive vagal stimulation methods such as transcutaneous vagus nerve stimulation. Where breathing training is typically administered prior to cognitive testing, transcutaneous vagus nerve stimulation is typically administered during task performance and is associated with improved performance in executive function tasks (Colzato & Beste, 2020; Ridgewell et al., 2021). Taken together with findings suggesting HRV increases do not extend beyond the active period of slow-paced breathing, other ‘online’ methods such as transcutaneous stimulation may better elucidate the influence of vagal activation on cognitive control in GAD.

There are notable strengths to the present study. It has previously been suggested that null effects of slow-paced breathing on post-training cognitive/HRV indices may have occurred because participants were breathing at a generic slow rate, as opposed to their own resonance frequency (Hoffmann et al., 2019; Laborde et al., 2022a). Indeed, some initial evidence suggests that breathing at resonance frequency induces significantly greater improvements to state mood and the LF/HF HRV ratio as compared with a rate of one breath per minute above resonance frequency (Steffen et al., 2017). In the present study, participants in the experimental group were asked to breathe at approximately their individual resonance frequency, following the recommendation and procedures of established protocols (Lehrer et al., 2000, 2013).

Another important methodological strength was the use of an active, comparative control condition which similarly individualised the breathing pace administered to each participant. Here, participants were tasked with breathing at approximately their mean respiration rate as measured at the beginning of the session. Previous research has often employed control conditions in which participants are not tasked with focusing on and controlling their breathing in a similar manner to the experimental breathing manipulation. For example, in previous single-session research, control participants have been asked to watch television (e.g., Hoffman et al., 2019; Laborde et al., 2019, 2022a; You et al., 2022, 2024) or sit quietly with no breathing instruction (Chelidoni et al., 2020; Steffen et al., 2017). These approaches therefore vary in the degree of cognitive and physiological effort expended between conditions. Alternatively, other studies have utilised breathing rates close to the range of resonance frequency, and therefore lower than normal human respiration (9 breaths per minute) (Meule & Kübler, 2017), or at the lower end of normal human respiration (12 breaths per minute) (Van Diest et al., 2014). In the present study, participants in both the experimental and control condition received the same instructions at each stage of the session and were presented with the same pacer stimulus during breathing practices, limiting confounding effects of external emotional valence or cognitive demand (e.g., relaxation or attentional distraction) on HRV change. Importantly, the control condition demonstrated significantly lower HRV indices than the experimental group within these parameters.

An additional strength was the use of a respiration monitor to track breathing rates across the phases of the experimental session, per Shaffer and Meehan’s (2020) recommendations. Observing mean breathing rates during paced breathing practice allows researchers to state with greater certainty the extent to which participants were able to accurately undertake the paced breathing. In the present study, our data demonstrated that our control group were breathing at a significantly faster rate than the experimental group, and that each group were breathing within expected ranges, overall (approx. 4–8 breaths in the experimental group and 12–20 breaths in the control group). Although adherence to slow-paced breathing manipulations can be inferred from increases to corresponding HRV indices (i.e., significantly higher LF-HRV during RFB), our approach allows us to suggest that participants did generally successfully follow the breathing pacer within each group. Therefore, the lack of expected between-group differences in post-breathing training performance in the present study is unlikely to be due to inefficacy of the experimental manipulation.

There are also limitations to the present study to consider. Data were collected across two testing sites and consequently the colour-word Stroop and emotional Stroop tasks were presented in two different languages (English in the London sample, Italian in the Rome sample). Words were matched for length and frequency in the English-language version of the task. However, words were translated directly into Italian and therefore it is possible that these words were not of equal emotional valence in both languages. This may have introduced variation in our data. However, we saw the same pattern of results across all tasks, including in the SART which required no translation, and the colour-word Stroop which had no specific emotional component. We also saw no significant differences in performance by site. Another potential limitation lies in the placement of the breathing training within the session. We employed three cognitive tasks in order to determine whether RFB has an influence on any specific aspect of inhibitory control (stimulus, emotional, or response inhibition). However, we did not ask participants to engage in breathing exercises directly before each task and therefore potential effects on the second and third task may have been washed out.

As noted, the 5-min breathing training periods used in the present study did not result in sustained change to HRV. Therefore, the breathing training periods were possibly not long enough to affect cognition. Future research could usefully incorporate longer breathing periods within an expanded design which includes participants reporting low levels of GAD symptoms as controls. From the present study, we can conclude that RFB did not improve inhibitory control in high GAD scorers, but further context is required to determine if these non-significant effects were specific to these high scorers, or alternatively whether the manipulation would have been beneficial for those beginning at a less cognitively and physiological restricted baseline (i.e. low anxiety scorers). Although short breathing training durations have been associated with improved cognitive performance (Bahameish & Stockman, 2024; Bonomini et al., 2020; Laborde et al., 2022a), very little is currently known about the dose–response response relationship of slow-paced breathing for reducing symptoms associated with GAD. While increased symptom severity is associated with increased likelihood of treatment response to cognitive interventions, having an anxiety disorder conversely decreases these odds (Andersson et al., 2019). Thus, comparisons of breathing manipulations across participants who vary in levels of self-reported symptoms presents an important focus for further study. Future research could also examine longer breathing training durations in this context. One possibility is that training over a number of weeks might produce a sustained rather than acute change in HRV, where effects of HRV increase on cognition could then be examined (e.g., Yoo et al., 2023; Nashiro et al., 2024 in individuals without pathological anxiety). However, there is currently limited evidence that training spanning weeks and incorporating twice-daily breathing practice has reliable effects on inhibitory control in a non-selected sample (Nashiro et al., 2023). The present work with cohorts experiencing high levels of anxiety where inhibitory control may be particularly impaired warrants further investigation with a higher ‘dose’ of RFB to sustain HRV increases.

## Conclusions

RFB successfully increased HRV indices in a participant sample reporting high levels of GAD symptoms, as compared with breathing at mean respiration rate. However, these increases in HRV were localised to performance of the breathing exercise: HRV did not significantly increase between baseline performance of inhibitory control tasks and a post-breathing administration of the task. In line with the non-significant changes in HRV following breathing training, behavioural inhibitory control did not improve from baseline to the second task administration. Our results suggest short RFB training can cause transient changes in HRV in participants reporting high levels of GAD symptoms. However, future research should include participants reporting comparatively low levels of GAD as a comparison, to determine whether these effects are generalisable. Future research should also investigate whether longer, more intensive training successfully sustains HRV increases in these groups, to better determine the impact of increased HRV on inhibitory control and broader cognitive function.

## Supplementary Information

Below is the link to the electronic supplementary material.

Supplementary file1 (DOCX 60 kb)

## Data Availability

The study was preregistered on the Open Science Framework prior to analysis of data, where the deidentified data for the study are available (https://osf.io/dut8v/).

## Footnotes

1 Task developed by Professor Colette Hirsch and team, as yet unpublished.

2 Per our preregistered analysis plan, follow-up analyses were planned to assess whether HRV mediated the relationship between group allocation and inhibitory control. However, as there were no significant associations between these variables, the mediation analyses were not conducted.
