# Supplementary material for: Effects of A Brief Resonance Frequency Breathing Exercise on Heart Rate Variability and Inhibitory Control in the Context of Generalised Anxiety Disorder
Source: Appl Psychophysiol Biofeedback. 2025 Feb 9;50(2):213–33. doi: 10.1007/s10484-025-09687-0 (PMC12081530; doi:10.1007/s10484-025-09687-0)
Supplement: Supplementary file 1 — Supplementary file1 (DOCX 60 kb) [file 10484_2025_9687_MOESM1_ESM.docx]

**Supplementary Materials: Effects of Resonance Frequency Breathing on Heart Rate Variability and Inhibitory Control in the Context of Generalized Anxiety Disorder.**

**Supplement 1. Power Considerations**

A meta-analysis (Lehrer et al., 2020) examining the effect of heart rate variability biofeedback training on executive function (EF) tasks reported an effect size of small – medium (Hedges *g* = .30) across studies. However, this is on a range of different EF tasks and study designs. Examining clinically meaningful change on an executive function task e.g. differences on the Rules Shift Card Test between those with (*N* = 30) and without (*N* = 30) an anxiety disorder indicated a large effect size (*d* = 1.48 95% CI -2.05, -0.90; Monteiro & Flávia, 2018). Based on these mixed prior findings we powered the study on the basis of a medium effect size (*d* = 0.5), requiring a minimum of 64 participants per group (*N* = 128). It should be noted that this is based on looking at differences between two groups on EF task performance and does not take into account within-group changes as examined in our mixed model group x time design and thus is in keeping with the aim to take a conservative approach to power.

**Supplement 2. Visual Analogue Scale Scores During the Experiment**

**Table S2**

*Visual Analogue Scale Scores From 0 - 100*

|  |  | **Control** | | |  | **Experimental** | | |
| --- | --- | --- | --- | --- | --- | --- | --- | --- |
| **Time** | **Question** | **Mean (SD)** | **Min - Max** | **Median** |  | **Mean (SD)** | **Min - Max** | **Median** |
| VAS 1 | Anxious | 49.3 (25.9) | 0 - 87 | 58.0 |  | 44.5(25.4) | 0 - 97 | 52.5 |
| VAS 2 | Anxious | 61.6(24.5) | 3-100 | 65.0 |  | 57.8(22.5) | 5-99 | 60.0 |
| VAS 3 | Anxious | 42.5(26) | 0-95 | 44.5 |  | 43.7(25.7) | 0-100 | 43.5 |
| VAS 4 | Anxious | 51.6(25.1) | 1-100 | 56.0 |  | 48.5(25.9) | 0-100 | 52.0 |
| VAS 5 | Anxious | 72.5(23.2) | 0-100 | 80.0 |  | 66.6(19.4) | 10-100 | 69.0 |
| VAS 1 | Worried | 41(25.5) | 0-86 | 45.0 |  | 40(25.5) | 0-87 | 42.0 |
| VAS 2 | Worried | 51(26.5) | 0-100 | 56.0 |  | 44.3(26.5) | 0-97 | 45.0 |
| VAS 3 | Worried | 38.9(26.6) | 0-95 | 45.0 |  | 40(25.5) | 0-92 | 50.5 |
| VAS 4 | Worried | 43.2(26.6) | 0-98 | 48.5 |  | 40.8(24.4) | 0-100 | 40.0 |
| VAS 5 | Worried | 77.9(18.5) | 5-100 | 81.0 |  | 71.1(17.6) | 28-100 | 71.0 |
| VAS 1 | Happy | 47.6(23.2) | 0-100 | 50.0 |  | 47.6(24.1) | 0-95 | 51.0 |
| VAS 2 | Happy | 36.2(22.4) | 0-100 | 40.0 |  | 36.4(21.8) | 0-90 | 40.0 |
| VAS 3 | Happy | 37.1(22.5) | 0-85 | 40.0 |  | 36.7(22.6) | 0-96 | 40.0 |
| VAS 4 | Happy | 33.2(22.4) | 0-81 | 39.0 |  | 35.2(22.7) | 0-90 | 40.0 |
| VAS 5 | Happy | 24.7(23.5) | 0-94 | 22.0 |  | 25.9(18.8) | 0-67 | 24.0 |
| VAS 1 | Sad | 24.2(22.3) | 0-74 | 20.0 |  | 28.3(24.9) | 0-98 | 23.5 |
| VAS 2 | Sad | 27.2(23.1) | 0-70 | 22.0 |  | 28(23.3) | 0-85 | 20.5 |
| VAS 3 | Sad | 24.2(21.6) | 0-65 | 17.0 |  | 28.8(24.6) | 0-100 | 26.0 |
| VAS 4 | Sad | 24.4(22.2) | 0-60 | 19.0 |  | 25.7(22.3) | 0-70 | 20.0 |
| VAS 5 | Sad | 56.2(29.2) | 0-100 | 63.0 |  | 58.2(23.7) | 0-100 | 60.0 |

*Note*. 1) Anxious = ‘Right now/During the task, how much do/did you feel anxious?’, Worried = ‘Right now/During the task, how much do/did you feel worried?’, Happy = ‘Right now/During the task, how much do/did you feel happy?’, Sad = ‘Right now/During the task, how much do/did you feel sad?’ 2) VAS 1 = Pre-baseline measures, VAS 2 = Post-baseline tasks, VAS 3 = Post-breathing training 1, VAS 4 = Post-second administration of cognitive tasks, VAS 5 = Post-behavioural worry task

**Supplement 3. Control Variables Analyses**

A Pearson’s chi-square test indicated that gender frequency did not vary significantly between groups, *χ^2^*(2) = 3.57, *p* = 0.17*.*  Then, logistic regression indicated that the experimental group and control group did not significantly differ in diagnoses of an anxiety disorder, use of hormonal contraceptives, over-the-counter medication, SSRIs, alcohol, caffeine, nicotine, and recreational drugs (all *p* > .20). Ordinal regression indicated that participants did not significantly vary in previous night’s sleep quality (‘very poor’, ‘poor’, ‘fair’, ‘good’, ‘very good’), odds ratio =0.71, 95% CI [0.38, 1.32], *t* = -0.43, and self-reported fitness levels (‘unfit’, ‘average fitness’, ‘fit’, ‘very fit’), odds ratio =0.87, 95% CI [0.45, 1.67], *t* = -1.07. As all of the above differences were non-significant, we did not include control variables in subsequent analyses.

**Table S3**

*Control Variables Descriptive Statistics*

|  | | **Overall**  **(*N* = 135)** | **Control**  **(*N* = 69)** | **Experimental**  **(*N* = 66)** |
| --- | --- | --- | --- | --- |
| **Hormonal contraceptive usage Yes/No (% responded ‘yes’)** | | 27 (20%) | 14 (20.3%) | 13 (19.7%) |
| **Regular practice of breathing exercises Yes/No (% responded ‘yes’)** | | 20 (14.8%) | 8 (11.6%) | 12 (18.2%) |
| **Caffeine consumption Yes/No (% responded ‘yes’)** | | 95 (70.4%) | 48 (69.6%) | 47 (71.2%) |
| **Over the counter medication Yes/No (% responded ‘yes’)** | | 15 (11.1%) | 10 (14.5%) | 5 (7.6%) |
| **Alcohol** | |  |  |  |
|  | No use | 39 (28.9%) | 21 (30.4%) | 18 (27.3%) |
|  | Slight use | 80 (59.3%) | 42 (60.9%) | 38 (57.6%) |
|  | Great social use | 16 (11.9%) | 6 (8.7%) | 10 (15.2%) |
|  | Abuse | 0 (0%) | 0 (0%) | 0 (0%) |
|  | Severe abuse | 0 (0%) | 0 (0%) | 0 (0%) |
| **Drug use** | |  |  |  |
|  | No use | 127 (94.1%) | 65 (94.2%) | 62 (93.9%) |
|  | Slight use | 8 (5.9%) | 4 (5.8%) | 4 (6.1%) |
|  | Great social use | 0 (0%) | 0 (0%) | 0 (0%) |
|  | Abuse | 0 (0%) | 0 (0%) | 0 (0%) |
|  | Severe abuse | 0 (0%) | 0 (0%) | 0 (0%) |
| **Nicotine use** | |  |  |  |
|  | No use | 96 (71.1%) | 52 (75.4%) | 44 (66.7%) |
|  | Slight use | 18 (13.3%) | 8 (11.6%) | 10 (15.2%) |
|  | Great social use | 14 (10.4%) | 7 (10.1%) | 7 (10.6%) |
|  | Abuse | 0 (0%) | 0 (0%) | 0 (0%) |
|  | Severe abuse | 0 (0%) | 0 (0%) | 0 (0%) |
| **Fitness** | |  |  |  |
|  | Unfit | 16 (11.9%) | 8 (11.6%) | 8 (12.1%) |
|  | Average Fitness | 75 (55.6%) | 37 (53.6%) | 38 (57.6%) |
|  | Fit | 43 (31.9%) | 24 (34.8%) | 19 (28.8%) |
|  | Very fit | 1 (0.7%) | 0 (0%) | 1 (1.5%) |
| **Quality of previous night’s sleep** | |  |  |  |
|  | Very poor | 2 (1.5%) | 1 (1.4%) | 1 (1.5%) |
|  | Poor | 28 (20.7%) | 11 (15.9%) | 17 (25.8%) |
|  | Fair | 53 (39.3%) | 28 (40.6%) | 25 (37.9%) |
|  | Good | 39 (28.9%) | 23 (33.3%) | 16 (24.2%) |
|  | Very good | 13 (9.6%) | 6 (8.7%) | 7 (10.6%) |

**Supplement 4. Heart Rate Variability Descriptive Statistics and Analyses During the SART Task**

**Table S4.1**

*RMSSD (log) and HF-HRV (log) During Each SART Administration*

Note: RMSSD = Root Mean Square Successive Differences metric of heart rate variability

HR- HRV = High frequency heart rate variability

|  |  | Control | | | | |  | | Experimental | | | | | |
| --- | --- | --- | --- | --- | --- | --- | --- | --- | --- | --- | --- | --- | --- | --- |
|  | Time | *N* | Mean | *SD* | Min | Max |  | *N* | | Mean | *SD* | Min | Max |  |
| **SART**  **RMSSD (log)** | 1 | 68 | 1.5 | 0.2 | 0.7 | 2.0 |  | 62 | | 1.6 | 0.2 | 1.1 | 2.2 |  |
|  | 2 | 66 | 1.6 | 0.2 | 1.0 | 2.1 |  | 62 | | 1.6 | 0.2 | 1.2 | 2.1 |  |
| **SART**  **HF-HRV (log)** | 1 | 68 | 6.1 | 1.2 | 2.5 | 8.6 |  | 62 | | 6.2 | 1.1 | 4.1 | 9.0 |  |
|  | 2 | 66 | 6.3 | 1.2 | 2.5 | 8.7 |  | 62 | | 6.3 | 1.1 | 4.4 | 9.0 |  |

**Table S4.2**

*Parameter Estimates for Each Linear Model Including RMSSD (log) and HF-HRV (log) during each SART Administration*

|  |  |  |  |  |  | *95% Confidence Intervals* | |
| --- | --- | --- | --- | --- | --- | --- | --- |
|  | *B* | *SE* | *df* | *t* | *p* | *LLCI* | *ULCI* |
| **RMSSD (log)** |  |  |  |  |  |  |  |
| Intercept | 3.52 | 0.06 | 144.05 | 56.12 | <.001 | 3.40 | 3.64 |
| Condition | 0.07 | 0.09 | 144.89 | 0.83 | .410 | -0.10 | 0.25 |
| Time | 0.16 | 0.03 | 124.78 | 5.40 | <.001 | 0.10 | 0.21 |
| Condition x Time | -0.08 | 0.04 | 125.02 | -1.83 | .069 | -0.16 | 0.01 |
| **HF-HRV (log)** |  |  |  |  |  |  |  |
| Intercept | 6.09 | 0.13 | 143.66 | 44.97 | <.001 | 5.83 | 6.36 |
| Condition | 0.13 | 0.20 | 144.49 | 0.67 | .507 | -0.25 | 0.51 |
| Time | 0.25 | 0.06 | 124.94 | 4.04 | <.001 | 0.13 | 0.37 |
| Condition x Time | -0.14 | 0.09 | 124.94 | -1.61 | .11 | -0.32 | -0.03 |

Note: RMSSD = Root Mean Square Successive Differences metric of heart rate variability

HR- HRV = High frequency heart rate variability
